# Cognitive Remediation and Psychosocial Rehabilitation for Individuals with Severe Mental Illness

**DOI:** 10.1155/2012/283602

**Published:** 2012-12-24

**Authors:** Susan R. McGurk, Shaun M. Eack, Matthew Kurtz, Kim T. Mueser

**Affiliations:** ^1^Center for Psychiatric Rehabilitation, Boston University, Boston, MA 02115, USA; ^2^Departments of Occupational Therapy, Psychology, and Psychiatry, Boston University, Boston, MA 02115, USA; ^3^Dartmouth Psychiatric Research Center, Dartmouth Medical School, Lebanon, NH 03766, USA; ^4^Department of Psychiatry, Dartmouth Medical School, Lebanon, NH 03766, USA; ^5^Western Psychiatric Institute and Clinic, School of Medicine, University of Pittsburgh, Pittsburgh, PA 15213, USA; ^6^School of Social Work, University of Pittsburgh, Pittsburgh, PA 15260, USA; ^7^Department of Psychology, Wesleyan University, Middletown, CT 06459, USA

Individuals with severe mental illnesses (SMI) such as schizophrenia are more likely to have impaired cognitive functioning in areas such as attention and concentration, psychomotor speed, memory, executive functions, and social cognition. These cognitive challenges are strongly associated with reduced psychosocial adjustment, such as the capacity for independent living, work or school, and social relationships, as well as the ability to benefit from rehabilitation programs targeted at improving outcome in these areas of adjustment. Cognitive remediation is the systematic use of methods aimed at improving cognitive functioning through the practice of cognitive exercises on either computer-based or paper and pencil tests, teaching more effective strategies for addressing cognitive challenges, and teaching coping or compensatory skills to reduce the effects of cognitive impairment on psychosocial functioning. 

For over 30 years, research has examined the feasibility and effects of cognitive remediation on cognitive functioning and psychosocial adjustment in people with SMI. Recent meta-analyses of controlled research have shown that cognitive remediation is associated with significant improvements in both cognitive functioning and psychosocial adjustment. However, these reviews of research have also found that the impact of cognitive remediation on psychosocial adjustment is contingent upon the provision of adjunctive or integrated psychiatric rehabilitation, such as supported employment or social skills training. That is, studies that have added or integrated cognitive remediation and psychiatric rehabilitation programs have been found to improve psychosocial functioning more than psychiatric rehabilitation alone, whereas studies that have added cognitive remediation to usual services have found little to no differences in psychosocial outcomes compared to usual services alone. This issue on cognitive remediation and psychiatric rehabilitation brings together six papers addressing topics critical to improving the long-term psychosocial functioning of people with SMI.

Four of these papers provide pertinent reviews of research that point the field in important new directions, and two papers are original research contributions. While the research suggests that combining cognitive remediation with psychiatric rehabilitation is more effective at improving psychosocial outcomes, very little is known about how such programs should be combined. The review by R. Penadés and colleagues addresses this question by providing a framework and hierarchical flowchart for integrating cognitive remediation with other evidence-based psychosocial interventions. D. L. Roberts and D. L. Velligan review relevant research suggesting that programs specifically developed for and targeting social cognition (e.g., emotion recognition, ability to infer other's mental states) may be more effective and efficient than broad-based cognitive remediation programs. This review has important implications for developing or refining cognitive remediation and psychiatric rehabilitation interventions in order to maximize their cost effectiveness. 

N. Contreras and colleagues provide a theoretical review of strategies for improving workforce participation in people with a SMI, with a particular focus on the Australian context. The review highlights the Individual Placement and Support (IPS) model of vocational rehabilitation, the most empirically validated program for improving employment outcomes in people with SMI, which has recently been combined with cognitive remediation in the USA. The authors suggest that Australia may be in a prime position to implement such combined interventions.

N. Boycott and colleagues also focused in their review on the IPS model, with a particular aim at evaluating the effects of supplementary interventions on work outcomes, including cognitive remediation. While prior research has evaluated the impact of adding cognitive remediation to vocational rehabilitation for people with SMI, scant attention has been paid to integrating cognitive remediation with supported education for this population. The paper by S. A. Kidd and colleagues addresses this lacuna by providing encouraging pilot data from such an integrated program, supporting both the feasibility of integrating cognitive remediation with supported education, and suggesting benefits in education-related outcomes. Finally, the research paper by M. N. Levaux and colleagues provides a useful case illustration of how cognitive remediation can be individually integrated and tailored into helping a person accomplish everyday life tasks. 

We believe that the papers compiled in this issue make an important contribution to the cognitive remediation field by providing useful syntheses of the research literature, suggesting new directions for research and clinical implementation, and presenting new data on promising programs and approaches to integrate cognitive remediation and psychiatric rehabilitation into people's daily lives.

*Susan R. McGurk* *Susan R. McGurk* *Shaun M. Eack* *Shaun M. Eack* *Matthew Kurtz* *Matthew Kurtz* *Kim T. Mueser* *Kim T. Mueser*

